# Impact of fear of COVID-19 on students' performance, moderating role of mindfulness: HSK students' perception-based view

**DOI:** 10.3389/fpubh.2022.967125

**Published:** 2022-11-07

**Authors:** Zhang Meiyi, Yang Liu

**Affiliations:** ^1^School of International Education, Shandong University of Finance and Economics, Jinan, China; ^2^Department of General Courses, Shanghai Information Technology College, Shanghai, China

**Keywords:** fear of COVID-19, students' performance, anxiety, mindfulness, stress theory

## Abstract

COVID-19 created difficulties and problems in almost everyone's daily life routine. Educational institutions too had to reschedule their academic activities. This shift caused attitudinal and behavioral changes in students' learning patterns. Using stress theory, the present study tries to determine the association of fear of COVID-19 with students' performance. In addition, the present study also attempts to check the impact of fear of COVID-19 on anxiety. Further, this study tries to find the association of anxiety with students' performance. This study also attempts to determine the mediating role of anxiety and the moderating role of mindfulness. For empirical investigation, the current study collected data from 320 HSK students from different colleges and universities in China. The present study applied partial least square structural equation modeling for the empirical investigation of hypotheses by using Smart-PLS software. The present study's findings confirmed that fear of COVID-19 negatively affects students' performance, and it positively correlates with anxiety. The study's outcomes revealed that anxiety negatively affects students' performance. The outcomes also confirmed that anxiety negatively mediates the relationship between fear of COVID-19 and students' performance. The present study's findings acknowledged that mindfulness does not moderate the relationship between fear of COVID-19 and student performance and has a positive moderation between anxiety and student performance. The present study offers important practical, theoretical, and managerial implications.

## Introduction

There is hardly any aspect of daily life that has not changed in the wake of COVID-19. The impact of the pandemic has been paramount. Students, like all other social groups, have had to cope with the challenges that the pandemic brought about (1). One of the biggest challenges for students and academic institutions was to continue the process of education while maintaining social distancing as required by governments (2). The use of online learning modules became a new normal with educational institutions switching from face-to-face learning to online learning methods, which ensured both synchronous and asynchronous communication between teachers and students (3). A variety of different technologies have been used by educational institutions to move from face-to-face learning to online learning. Video conferencing, instant messaging applications, and other educational online tools are a few of the examples that have been used to continue the process of education. Imparting education has somehow been managed by educational institutes with the help of technology; however, there has been a surge in interest in studies concerning how COVID-19 has been affecting the mental health of students. Far-reaching implications of online learning have been discussed widely and there has been concern regarding how this rapid and drastic change has affected the mental health of students (4). This particular study attempts to examine the extent and nature of this effect on the mental health of students.

Studies have tried to examine the extent of fear of a pandemic, quarantines, and economic hardships that have generally been considered problematic, particularly among students (5). Some studies found that students faced tremendous anxiety and stress as they lacked access to either internet or the required technology. It was also found that some educational institutes were not able to adapt to the modalities which caused students to fear the loss of the school year (6). The fear of contracting the virus constrained the students in their homes as social distancing had to be ensured. On the other hand, the distress of inefficient learning on account of multiple factors compounded the intensity of fear for students. The students who were not in a position to afford these modalities were more prone to experience stress and anxiety (7). Furthermore, it was also perceived by the students that they have to put in extra effort to learn using these modalities, and teachers were not totally at ease using these modalities to impart knowledge properly while they gave lessons (8). Knowledge, expertise, and aptitude indicate the academic performance of a particular student in a certain academic field. These indicators are evaluated through grades that the students secure for different subjects that are part of a study plan. Nevertheless, the examination of a student's knowledge and aptitude is not a matter of straightforward scrutiny merely based on a student's grades (9). Many different factors can negatively affect the performance of a student. For instance, high levels of anxiety, depression, students' negative attitude toward academic endeavors (10), prolonged use of the internet, lack of quality sleep (11), poor learning plans (12), and consumption of alcohol or other drugs (13, 14) can have an adverse impact on academic performance.

Fear of COVID-19, losing academic prospects, and inability to cope with an array of surging pressures negatively impacted the mental health of students. This study aims to explore the extent of the negative impact of COVID-19, and how it has impacted the mental health of the students. There has been overwhelming evidence that COVID-19 has impacted many students in a very negative way as many started to show symptoms of poor mental health and those who already had a history of mental illness saw their situation exacerbate even further (15–17). Fear, depression, anxiety, and irregular sleep patterns were the main problems found particularly in those who had contracted COVID-19 (18–20). Among the people in Wuhan, a Chinese city from where the coronavirus spread, those who already had mental health issues were highly vulnerable to the risk of further deterioration of their mental wellbeing (21). The number of positive coronavirus patients in China who already had psychiatric disorders saw a significant surge in anxiety and fear (22). Quarantine was the most commonly used preventive measure during the pandemic; however, the impact of isolation on mental wellbeing has not been very encouraging. Anxiety and depression caused by quarantine were obvious in most studies (23).

Many studies indicated that the students were not particularly enthusiastic about online learning as they preferred face-to-face learning (24). The idea of virtual education met with less enthusiasm from the students who reported a lack of motivation while studying online (25, 26). Such negative perceptions regarding online learning, theoretically, can hamper the academic performance of students (7, 27, 28). Contrarily, some of the students have shown improvement in their academic performance and seem to have felt comfortable with the new and advanced methods of learning (29).

The *Hanyu Shuiping Kaoshi* (HSK, Chinese Proficiency Test) students have not been an exception during this pandemic as they too faced multiple issues regarding learning. Therefore, it is imperative to examine the factors that are more influential in impacting the academic performance of HSK students during the pandemic. Moreover, it is also important to study if these factors are responsible for improving or decreasing the academic performance of these students. During the pandemic, many studies have been carried out on various types of students using various research models, but no study has focused on HSK students as a subject of research. This has created a study gap that needs to be filled. The objective of the current study is to fill this gap by studying the impact of fear of COVID-19 on the academic performance of HSK students and exploring the moderating role of mindfulness to overcome this fear. The current study aims to examine the following four parameters using the lens of stress theory. Firstly, it examines the relationship between fear of COVID-19 and anxiety. Secondly, the relationship between fear of COVID-19 and students' performance is assessed. Thirdly, it studies the relationship between anxiety and students' performance. Fourthly, the study examines the facilitating role of mindfulness in fear of COVID-19 and students' performance.

The novelty of this research study lies in the selection of study parameters and research subjects. It serves the literature in the following ways. First, it explores and presents the available scientific literature on stress theory, modes of education during the pandemic, the mental health of students, and the mediating role of mindfulness to improve mental health. Furthermore, it provides novel scientific data on the perception-based study of variables such as fear, anxiety, and mindfulness.

## Literature review

COVID-19 has become the most feared virus since it first emerged in Wuhan, China in December 2019. Depression, anxiety, and fear are the most common psychological symptoms that COVID-19 has caused worldwide (30). The US and other European countries such as the United Kingdom, Spain, Italy, and Portugal had to fight the serious repercussions of COVID-19 throughout the year 2020 (31). However, 2021 was the most difficult year for many Asian countries such as India, Philippines, Indonesia, Vietnam, and Thailand that were caught in the wave of surging death toll and virus infections. Symptoms of anxiety and depression rose significantly because of COVID-19 as people had to bear strict lockdowns and social distancing norms enforced by different governments throughout the world to contain the virus (30). One of the core mental health problems associated with the COVID-19 virus was the rise of fear in many people.

### Theoretical support

Anything posing a threat to our wellbeing is stressful. Stress probably is a good thing for the human race to inch forward in its bid to develop as without stress life may become boring and pointless. However, stress causing harm to our psychological and physical wellbeing is harmful stress. There are various forms of stress that students endure ranging from the stress to excel academically and the stress of graduating, and from the stress of an uncertain future to the stress of finding a foothold in the job market. The learning ability of a student also heavily depends on many factors such as social, emotional, and physical factors (32). Stress can lead to serious mental as well as physical health issues, decrease self-esteem, and ultimately affect the academic performance of the students (33). The impact of stress on students' learning capacity has received immense attention recently within the education system. Stakeholders such as teachers and parents tend to undermine the role of stress in an academic setup by comparing current experiences with their own experiences at school or college learning was probably not that stressful (34).

However, the experience of today's students attending college is often stressful and unsatisfying. Stress is caused by many factors such as the pressure to secure good grades, the urge to perform well, career aspirations, and the campus environment. Before being highly critical of the stress from the start, there is a need to acknowledge that stress is bad only when it is in excess. Mostly, stress stimulates our urge to perform better and improve our abilities. Challenging life events entail stress; however, the issue becomes serious when stress becomes overwhelming. Although, sometimes stress results from deeper and more serious emotional issues, however, mostly it is not significantly serious and normal counseling or incorporation of stress-management techniques work well to mitigate the stress.

Research suggests that individuals can appraise stressful events as threatening or challenging (35). In the wake of academic stress, students work more efficiently and feel a sense of achievement as their ability to learn increases as a result of the impetus provided by the stress. However, feelings of helplessness and a sense of loss become apparent when there is educational pressure. An important issue regarding stress among students is how it affects their learning. One study hypothesized that both low and high stress leads to learning deficiency, whereas moderate stress helps boost the learning process (36). Field studies and laboratory tests support this notion that high stress affects a student's performance negatively. Meeting deadlines and intending to perform well in exams are the core reasons for students to be stressed. Fear of failing, overwhelming workload, or peer competition are a few other reasons that cause stress among students (22). The period just before the start of exams is considered to be a period of high stress. Studies support performance deteriorates under stress (37), and it has been established that there is a correlation between stress and poor academic performance. It is argued that it is primarily undergraduate students who face the negative effects of stress as they are under pressure to do well academically because it is linked to their future job prospects.

### Relationship of fear of COVID-19 with anxiety and academic performance of students

An unpleasant emotional state provoked by a perceived threat is called fear (38). With the pandemic and lack of an effective treatment or cure, people feared developing symptoms of the disease or associated death caused by COVID-19 (5). It is referred to as fear of COVID-19 (39), and studies have been carried out globally to study the impact of this fear on individuals (40). Various research shows that the fear of COVID-19 has the potential to evolve into many different mental health issues (41). Some of the issues that were reported to have developed due to fear of COVID-19 include psychological distress (42), insomnia, post-traumatic stress symptoms, and particularly moderate to severe depressive symptoms and anxiety (43). Individuals who faced life-threatening scenarios were more prone to develop depression and anxiety. Additionally, anxiety and stress are also outcomes of mental fatigue caused by lockdowns, quarantines, strict adherence to social distancing, and the fear of contracting the virus itself (44).

While the fear of COVID-19 has a strong association with anxiety and stress, and this in turn leads to low or poor academic performance, it impacts academic performance in particular ways. There are four categories of fear that results from COVID-19 anxiety that have a profound impact on the academic performance of the students. First is the fear of wasting precious money on acquiring online education which to some is not as effective as face-to-face academic sessions (45). Second, loneliness and inactivity turned into a kind of fear among students who lived under strict lockdowns with confined movements and social interactions (46). Third, is fear of distraction during the process of online learning (47). Fourth, is the fear of lack of access to facilities such as computers and internet connections to attend online sessions effectively (48). There is concrete evidence that fear of COVID-19 has been a huge hindrance in the learning process of students, with anxieties and stresses of multiple kinds compounding the negativity around online learning. It is similar to a condition in which there are limited resources for learning, thereby hampering the learning process (49). Thus, based on the above literature, this study hypothesized:

***H1:*** *Fear of COVID-19 has a negative association with students' performance*.
***H2:*** *Fear of COVID-19 has a positive association with anxiety*.

### Relationship of anxiety academic performance of students

Impairment of function and substantial distress can lead to anxiety which is a physiological disorder. Anxiety is experienced when thoughts and feelings create an impression of an inability to handle or predict future events. Additionally, vague fear and impulses that are not known are defined as anxiety. Raised heartbeat, sweating of the palms, or insomnia are the few symptoms of anxiety. Anxiety has been identified as having two different characteristics such as state anxiety and trait anxiety (50). In trait anxiety, the perception of fear remains absent in the presence of a threat. Whereas, on the contrary, state anxiety is identified when an individual believes that a threat would harm him/her negatively. Simply put, state anxiety occurs when the threat of harm is felt by the individual. Generally, state anxiety is subjective, and is characterized by a constant presence of doubt created by the nervous system. It continues to change with time as the person perceives the idea of threat in the wake of stimuli or the imagination of the presence of a particular stimulus. Being subjective, the reaction to a stimulus differs based on the particular individual's level of threat perception.

Previous research has established that anxiety and academic achievements are closely related. Students who suffer from an anxiety disorder or any other kind of fear do not perform as efficiently as they would have liked, both in their personal and academic life. For instance, one study that examined the relationship between anxiety and academic performance (51), found that observation, problem-solving capability, learning capacity, and retaining information were badly affected by anxiety. The research concluded that anxiety lowered the level of academic performance of students. In general, anxiety triggers confusion and a high level of difficulty in comprehension, and the higher the anxiety, the lower the academic performance. Many other studies equally support the hypothesis that academic performance does get negatively impacted because of anxiety. For instance, a significant negative correlation was found between academic performance and anxiety (52). Another study found that as anxiety increased, academic achievement decreased, particularly because of the undermining of students' cognitive functions due to anxiety (52).

One of the factors that has an impact on a student's performance is the perception and experience related to an academic event or a particular subject that induces anxiety. For instance, according to a study (53), if a student has a negative experience with taking a mathematics test (e.g., failing the test or securing poor grades), the student tends to develop higher levels of anxiety whenever the mathematics test is to be taken and, in turn, this bad experience triggers a high level of anxiety leading to poor performance. Moreover, perception and experience related to a particular academic event can also induce anxiety. Such as, if a student experiences some sort of negativity vis-à-vis a mathematics test, the student will most likely develop an intense level of anxiety whenever a mathematics test has to be taken again, and the previous bad experience is likely to further increase anxiety in the student ultimately lowering the student's academic performance (54). Conversely, if a positive experience has already been experienced by an individual, then going back to repeat the same activity does not trigger anxiety which translates into better performance. Therefore, when analyzing anxiety's relationship with the performance it is important to take cognitive assessments, motives, and tendencies as well as previous experiences into consideration (55). Ultimately, anxiety's effect is equivocal. If anxiety level is significantly high, it is likely to influence academic performance negatively. However, low anxiety is likely to work as a motivating force to perform well.

Fear of COVID-19 is related to anxiety and stress, and it is also associated with depression but to a lesser extent. Even though there has been a lower association between depression and fear, cases of suicides have been reported due to COVID-19-related fear and stress (56). Furthermore, information overload regarding the increase in the number of COVID-19 cases and the presence of COVID-19-related news in media acted as stimuli to cause mood disorders in students (57). That is perhaps why researchers have found a higher level of anxiety and stress among the Chinese student population.

Gender and age-related differences in experiencing anxiety have come to the fore in research in the wake of the COVID-19 crisis. Women and younger people are more prone to be affected negatively by COVID-19 in terms of anxiety, stress, and depression (58). Nevertheless, most of these studies were carried out with a sample population related to healthcare setups. Moreover, the impact of the pandemic is understudied in younger people. It was found that undergraduates are more prone to get fearful of COVID-19 as compared to graduates in some studies. Strict lockdowns and social distancing regimes put in place by the respective governments were found to be the core cause of symptoms of anxiety and depression among young students (59). Thus, based on the above literature, this study hypothesized:

***H3:*** *Anxiety has a negative association with students' performance*.
***H4:*** *Anxiety negatively mediates the relationship between fear of COVID-19 and students' performance*.

### Mindfulness and its moderating role

Mindfulness is defined as “the awareness that arises when paying attention on purpose, in the present moment, and non-judgmentally” (60). There are three characterizing elements in this definition. Firstly, the ability to pay attention to a particular experience while being completely aware of it. Secondly, the process of bringing attention to the experience of the present moment, particularly without judging the experiences as good or bad. Thirdly, awareness of the results that arise from the process of paying attention. Awareness in the context of Mindfulness-Based Stress Reduction (MBSR) and Mindfulness-Based Cognitive Therapy (MBCT) is called mindful awareness (61). The practice of mindfulness and its benefits are quite elaborate. In this study, the most relevant benefits of mindfulness are mentioned within the context of the paramount challenges of anxiety and stress posed by the COVID-19 pandemic. There are five mindfulness skills such as observing, non-judging, non-reacting, acting with awareness, and describing.

Observing is about paying deliberate and close attention to the day-to-day experiences such as sitting, standing, walking, showering, or sensory stimuli related to sound, sight, and smell. Non-judging is the tendency to properly analyze one's experience, feelings, thoughts, and emotions to know if they are irrational, bad, or inappropriate. Moreover, it is also the ability to analytically observe and assess oneself. Non-reactivity is the ability to know distressing feelings and thoughts without being influenced by them. Such thoughts and feelings are assessed in a more decentralized way. Unwanted feelings and thoughts resulting from unwanted experiences are left to pass. Acting with awareness gauges the inclination of getting distracted from the present-moment experience by letting the mind wander, going on an auto-pilot mode, or quickly performing the activities without paying deep attention to them. Describing is the ability to explain one's feelings, thoughts, beliefs, sensations, and opinions in words. However, it is still being debated whether the ability to act with awareness could be captured by its opposite, which is the lapse of attention. Similarly, whether the ability to describe is a core mindfulness skill or not continues to be debated (62). Nevertheless, it has been demonstrated that skills that are learned through mindfulness practices are far more efficacious in controlling issues such as depression, fear, post-traumatic stress, anxiety, and eating disorders by putting them onto cognitive and effective concepts of the Research Domain Criteria Matrix that are adopted by the NIH (National Institute of Health) (63).

Positive psychological strengths are critical to countering the negative effects of fear during adverse situations as they lessen the psychological burden of prolonged distress (64). In this regard, one of the important concepts that might help to cope with such fear is mindfulness. It refers to the awareness of the present moment and acceptance of thoughts and feelings without judgment (65). The literature suggests that mindfulness is associated with an attitude of acceptance of difficult circumstances and emotions, which in turn facilitates effective responses to stressful stimuli (66). Thus, numerous clinical and experimental studies have supported the idea that mindfulness enhances positive psychological strengths such as resilience to stress, life satisfaction, bravery, self-regulation, and self-compassion (67). Moreover, individuals with high levels of mindfulness are likely to possess psychological strength that is important in countering the adverse effects of fear (64). In this regard, one of the probable remedies that might help to handle such fear is mindfulness. It refers to the cognizance of the existing moment and acceptance of feelings and moods without judgmental instincts (63). The literature proposes that mindfulness is related to an attitude of reception of tough situations and sentiments, which in turn eases effective responses to taxing provocations (66). Thus, several studies have maintained the idea that mindfulness increases positive mental strengths such as resilience, life gratification, courage, self-regulation, and self-compassion in the wake of anxiety (67). Moreover, distressing feelings caused by depression or anxiety are less likely to be affecting those people who are mindful in their approach. Studies have shown that mindfulness does decrease the individual's level of depression and mitigates anxiety symptoms (68).

Mindfulness, therefore, becomes very important, particularly during the times of extended distress of an ongoing pandemic that has changed the patterns of normal life as people have experienced prolonged periods of uncertainty, stress, and anxiety. As depression and anxiety can be mitigated using techniques of mindfulness, it is also likely that fear of COVID-19 can also be addressed through mindfulness (69). As mindfulness helps to maintain focus on the present moment and the fear of COVID-19 is mostly about the uncertainty surrounding the future and about events that have not even taken place, focusing on the present moment is the key to warding off the fear of COVID-19. Mindfulness can surely help one maintain one's mental health. There is, however, a pressing academic need to understand the impact of fear of COVID-19 that leads to stress and anxiety that ultimately translates into poor academic performance (44). Thus, based on the above literature, this study hypothesized hypotheses 5 and 6.

***H5:*** *Mindfulness positively moderates the relationship between fear of COVID-19 and student performance*.
***H6:*** *Mindfulness positively moderates the relationship between anxiety and student performance*.

**Figure 1:**
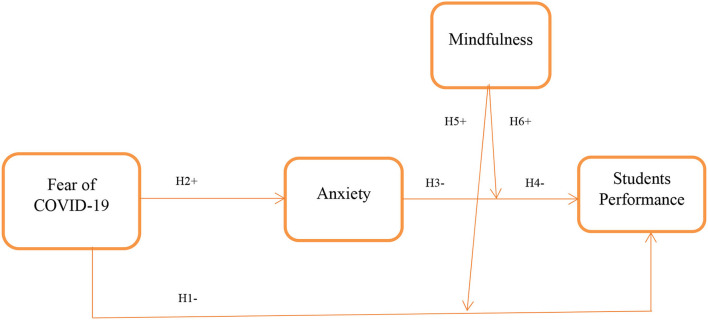
Conceptual framework.

The conceptual framework of this study is presented in Figure 1.

## Research methods

### Study design

The present study collected data from HSK students for the empirical investigation of the hypotheses. The convenience sampling technique was used for data collection from students (70). Prior studies of similar nature had collected data from students by adopting the online survey method during the peak of COVID-19 (71–73). Therefore, the present study preferred physical data collection and also because students were allowed to attend physical classes. For this purpose, the authors visited different colleges and universities regardless of social influence and met with the Heads of the HSK departments. The authors explained the whole study objective and requested their support for data collection. Additionally, the author ensured data confidentiality and assured them that it would be used only for research purposes. Further, the author promised to share the practical implications of this study at their request. Hence, the Heads of the departments permitted data collection. The target of the present study was to collect data from HSK students, so the authors developed a questionnaire in English and translated it into Chinese. For the language correction, senior researchers were consulted, and according to their guidance questionnaire was translated. Sample-based data was collected from the students as per the advice of the senior researchers, all errors were corrected, and the senior researchers approved the final version of the questionnaire. A cover note was attached with the questionnaire that explained the objectives of the present study, ensured the students of data privacy, and informed them that their individual-level responses would be destroyed while aggregated outcomes would be shared. In addition, this letter also assured the students that there were no right or wrong answers, and their unconstrained responses would be a great contribution to the successful accomplishment of this study instead of answers filled in consultation with others. This process was important to help enhance the confidence of the students and data reliability. In this way, students filled out questionnaires of their own will. The authors decided to collect data at different times as this step reduces the common method bias (74). Therefore, the time lag data method was adopted to fill out the questionnaires in four rounds. The questionnaires included a hidden code to recognize the responses of the same student. In the first round, 600 questionnaires regarding fear of COVID-19 among students were distributed, of which 509 valid and complete questionnaires were received. After a week's gap, in the second round, 509 questionnaires on anxiety were distributed and 428 complete and valid questionnaires were received. In the third round, after a further one-week gap, 428 questionnaires on students' performance was distributed and 389 valid and complete questionnaires were received. The fourth round with questionnaires on mindfulness was distributed after another one-week gap to 389 respondents of which 320 valid and complete questionnaires were received from the students. Hence, this study is based on a sample size of 320.

### Measures

This study used the five points Likert scale to measure the participants' responses, where 1 denoted “strongly disagree,” 2, “disagree,” 3, “neutral,” 4, “agree,” and 5 indicated “strongly agree.” This study assessed data from previously validated items.

#### Fear of COVID-19

The construct, “fear of COVID-19” was measured using a seven-item scale adapted from Ahorsu et al. (75). The items included statements such as, “When watching news and stories about coronavirus-19 on social media, I become nervous or anxious.”

#### Anxiety

The construct, “anxiety” was measured using a five-item scale adapted from Chen et al. (76) and included items such as, “I felt dizzy, lightheaded, or faint when I read or listened to news about the coronavirus.”

#### Mindfulness

The construct, “mindfulness” was measured using a five-item scale adapted from Chen et al. (77). It included items such as, “It seems I am running on automatic, without much awareness of what I'm doing.”

#### Student performance

“Student performance” was measured with a four-item scale adapted from Yousef et al. (78). This scale measured the comparative productivity of the students in their class, and included items such as, “To what extent do you agree that you perform better than your other class fellows.”

## Results

### Assessment of measurement and structural model (mediation)

The present study chose the variance-based partial least squares structural equation modeling (PLS-SEM) technique for analysis instead of other co-variance-based techniques such as AMOS (79). The main purpose behind this selection is the effectiveness of PLS-SEM for both confirmatory and exploratory types of studies (80). Structural equation modeling (SEM) comprises two different types, which include covariance-based (CB-SEM) and PLS-SEM (81). The key difference in both these methods is that while CB-SEM accepts or rejects theories, PLS-SEM aids in advancing and developing theories (82). PLS-SEM is a very suitable approach for complex and multi-order-based models. Furthermore, PLS-SEM is very useful for evaluating small data sets (82). Hence, the current study considered the PLS-SEM method for empirical data analyses using Smart PLS 3.3.3 software. The outcomes of PLS-SEM-based analysis were evaluated in two stages, including model measurement and structural model evaluation.

The model consisted of 16 reflective items of three variables (Table 1) and the results of the model measurement had two parts: model reliability and validity. The present study considered the values of “Cronbach's alpha, roh_A, composite reliability, and average variance extract (AVE)” to check the model's reliability (81), and all values are shown in Table 1. The values of Cronbach's alpha are accepted if they are greater than 0.7 (82). Similarly, the composite reliability values are considered good if >0.7. The Cronbach's alpha values of the models' constructs (anxiety, fear of COVID-19, and students' performance) were 0.870, 0.898, and 0.849, and the composite reliability values of models' constructs were 0.906, 0.920, and 0.895, respectively. All values of Cronbach's alpha and composite reliability were within acceptable standards, which confirm the model's reliability in the present study. The roh_A reliability values (0.872, 0.901, and 0.870) were also according to acceptable criteria (83). The average variance extract (AVE) values exceeding 0.5 are considered appropriate for the model's convergent validity (82). Table 1 illustrates that the AVE values (0.658, 0.623, and 0.682) were according to acceptable criteria.

**Table 1:** Reliability and convergent validity of the study constructs (mediation).

**Construct**	**Item**	**Outer loadings**	**VIF**	**Alpha**	**roh-A**	**Composite reliability**	**AVE**
ANX	ANX1	0.851	2.362	0.870	0.872	0.906	0.658
	ANX2	0.775	1.891				
	ANX3	0.811	1.981				
	ANX4	0.795	1.955				
	ANX5	0.821	1.969				
FOC	FOC1	0.802	1.257	0.898	0.901	0.920	0.623
	FOC2	0.756	1.923				
	FOC3	0.808	2.379				
	FOC4	0.849	2.965				
	FOC5	0.809	2.492				
	FOC6	0.788	2.083				
	FOC7	0.704	1.630				
SP	SP1	0.847	2.383	0.849	0.870	0.895	0.682
	SP2	0.839	3.325				
	SP3	0.856	3.508				
	SP4	0.757	1.292				

ANX, anxiety; FOC, fear of COVID-19; SP, student performance.

**Figure 2:**
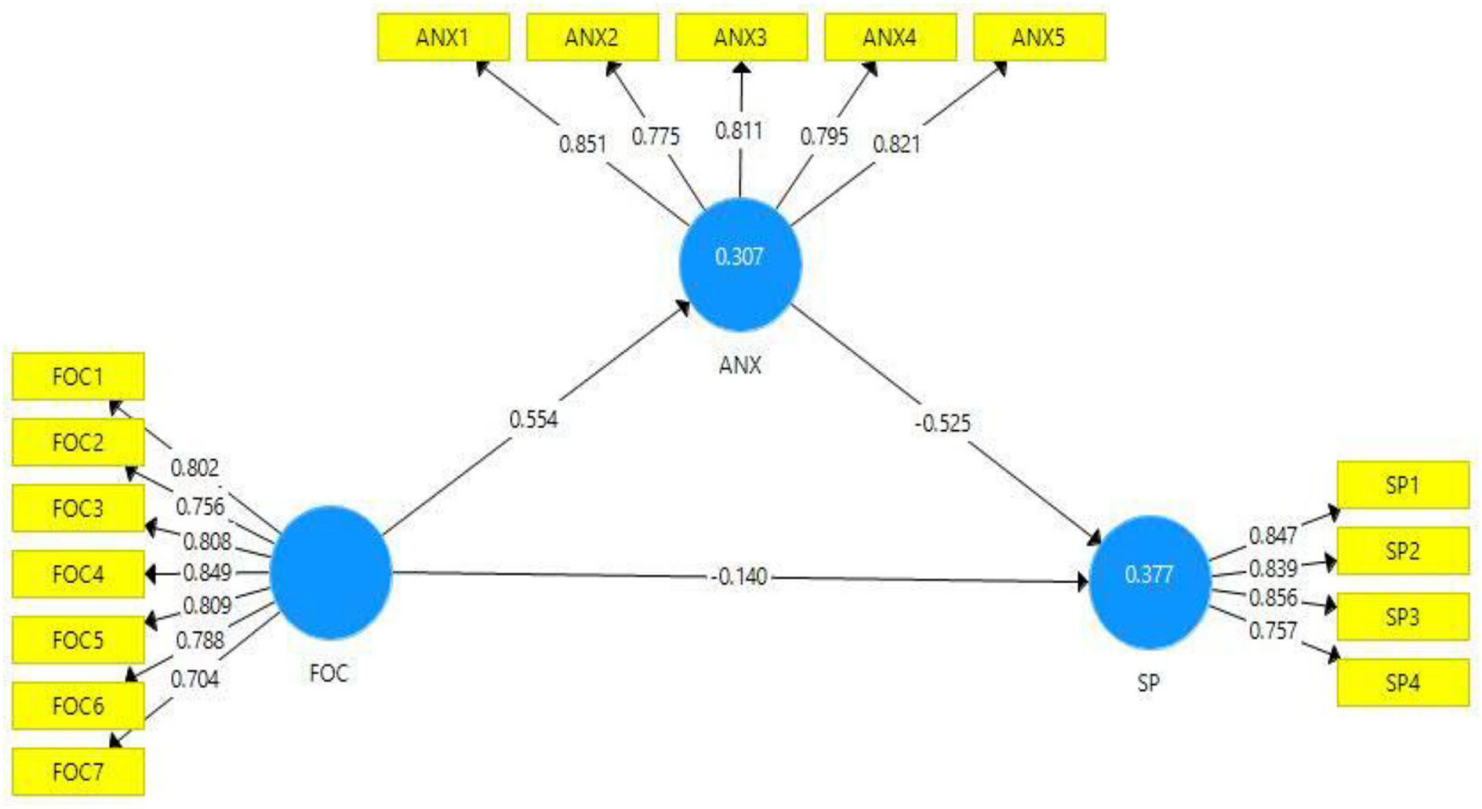
Path estimates and outer loadings (mediation).

All items' outer loading values of models' constructs are shown in Table 1. According to experts, the outer loading values ≥0.7 are considered reliable for the model's validity (82). Figure 2 describes that the outer loading values of all constructs' items are according to the required criteria. The variance inflation factor (VIF) values are also presented in Table 1. The VIF values are measured to validate the collinearity issues in the model. The model is considered free from collinearity issues if the VIF values are < 0.5 (79). According to the outcomes presented in Table 1, all VIF values were < 0.5, such as the variable “students' performance” item SP-3 has the highest VIF value (3.508). Hence, it is proven that there are no collinearity issues in the present study model.

The *R*^2^ values define the model's strength, such as the values of latent variables greater than or near 0.5 indicates moderate strength of the model, and the values near or below 0.25 show weak model strength (80). The *R*^2^ values of endogenous variables of the current study model (anxiety and students' performance) are 0.307 and 0.377, respectively, which shows moderate model strength. The cross-validated redundancy (*Q*^2^) values of the model are considered significant if they are larger than zero (80). The *Q*^2^ values of all latent variables of the present study are greater than zero, which demonstrates the significance of the model.

Two well-known approaches are used to approve the discriminant validity of the current study, namely, Fornell–Larcker criterion and heterotrait–monotrait (HTMT) ratios (79). The Fornell-Larcker criterion is assessed by taking the square roots of AVE values of model constructs (79). Fornell-Larcker criterion values of variables are presented in Table 2. The values under the Fornell-Larcker criterion are accepted if the upper side first value of each column is highest than their below values. Table 2 shows that all values of the Fornell-Larcker criterion are as per the required criteria. Thus, it is confirmed that discriminant validity based on the Fornell-Larcker criterion has been achieved in this study model. In addition, according to the given criteria, the HTMT values of all constructs should be < 0.85 though values >0.90 are also acceptable (83). According to the present study results, the HTMT values of constructs are < 0.85, which confirms that discriminant validity in the present study's model had been established (Table 3).

**Table 2:** Discriminant validity (Fornell-Larker-1981 criteria) (mediation).

**Construct**	**ANX**	**FOC**	**SP**
**ANX**	**0.811**		
**FOC**	0.554	**0.789**	
**SP**	−0.603	−0.432	**0.826**

ANX, anxiety, FOC, fear of COVID-19, SP, student performance. Bold values indicate the relationship and significance between the variables.

**Table 3:** Discriminant validity (HTMT) (mediation).

**Construct**	**ANX**	**FOC**	**SP**
**ANX**	–	–	–
**FOC**	0.626	–	–
**SP**	0.656	0.475	–

ANX, anxiety; FOC, fear of COVID-19; SP, student performance.

### Model estimation, direct and indirect (stage 1)

The empirical investigation of the current study was conducted by using a bootstrapping approach through 5,000 samples with replacements to estimate the significance level. The direct, indirect, and total paths are presented in Table 4. The present study considered the “*t*” values and “*p*” values of statistics for the acceptance or rejection of the hypotheses. The results of the current study's hypotheses are seen in Table 5. According to hypothesis 1, fear of COVID-19 negatively affects students' performance; the outcomes (*t* = 2.284, *p* = 0.022) showed that hypothesis 1 of the present study is accepted. In addition, the beta value of hypothesis 2 revealed that one unit change in the independent variable (fear of COVID-19) would result in 0.140 changes in the dependent variable (students' performance). The outcomes (*t* = 6.806, *p* = 0.000) of hypothesis 2 confirmed that the fear of COVID-19 positively correlates with anxiety, which means hypothesis 2 of the present study is also accepted. Additionally, the beta value of hypothesis 2 revealed that one unit change in the independent variable (fear of COVID-19) would result in 0.554 changes in the dependent variable (anxiety). According to the results (*t* = 8.502, *p* = 0.000) of the third hypothesis, anxiety negatively affects students' performance, which confirms that hypothesis 3 of the present study is also accepted. In addition, the beta value of hypothesis 3 showed that one unit change in the independent variable (anxiety) would result in 0.525 changes in the dependent variable (students' performance).

**Table 4:** Direct, indirect and total path estimates (mediation).

	**Beta**	**SD**	***t***	***p*-value**
**Direct path**				
**ANX → SP**	−0.525	0.062	8.502	0.000
**FOC → ANX**	0.554	0.081	6.806	0.000
**FOC → SP**	−0.140	0.062	2.284	0.022
**Indirect path**				
**FOC → ANX → SP**	−0.291	0.065	4.494	0.000
**Total path**				
**ANX → SP**	−0.525	0.062	8.502	0.000
**FOC → ANX**	0.554	0.081	6.806	0.000
**FOC → SP**	−0.432	0.082	5.249	0.000

ANX, anxiety; FOC, fear of COVID-19; SP, student performance.

**Table 5:** Hypotheses testing (mediation).

		**Coefficient (beta)**	**SD**	***t***	***p*-value**	**Status**
**Hypotheses**						
H1	FOC **→** SP	−0.140	0.062	2.284	0.022	Supported
H2	FOC **→** ANX	0.554	0.081	6.806	0.000	Supported
H3	ANX **→** SP	−0.525	0.062	8.502	0.000	Supported
**Mediation hypotheses**						
H4	FOC **→** ANX **→** SP	−0.291	0.065	4.494	0.000	Supported

ANX, anxiety; FOC, fear of COVID-19; SP, student performance.

The present study also considered the mediating role of anxiety between fear of COVID-19 and students' performance. For the empirical investigation of anxiety as a mediator, this study proposed hypothesis 4 (anxiety negatively mediates the relationship between fear of COVID-19 and students' performance). The results of the hypothesis testing (*t* = 4.494, *p* = 0.000) confirm that anxiety negatively mediates the relationship between fear of COVID-19 and students' performance. Additionally, the path value (0.291) of hypothesis 4 also confirms that anxiety negatively mediates the relationship between fear of COVID-19 and students' performance.

### Assessment of measurement and structural model (moderation analysis)

For a reflective measurement of the model, Smart-PLS recommends a two-stage method for moderation analysis, including “model measurement and model estimation” (82). The moderation analysis of the present study requires that all basic criteria (construct reliability and validity) and indicators of the model assessment such as out loading values, CR, Cronbach's alpha, rho_A, and AVE are according to acceptable criteria (80). Table 6 describes the particulars of the model assessment indicators.

**Table 6:** Reliability and convergent validity of the study constructs (moderation).

**Construct**	**Item**	**Outer loadings**	**VIF**	**Alpha**	**roh-A**	**Composite reliability**	**AVE**
ANX	ANX1	0.851	2.362	0.870	0.871	0.906	0.658
	ANX2	0.775	1.891				
	ANX3	0.811	1.981				
	ANX4	0.795	1.955				
	ANX5	0.821	1.969				
FOC	FOC1	0.802	1.257	0.898	0.901	0.920	0.623
	FOC2	0.756	1.923				
	FOC3	0.808	2.379				
	FOC4	0.849	2.965				
	FOC5	0.809	2.492				
	FOC6	0.788	2.083				
	FOC7	0.704	1.630				
MF	MF1	0.762	1.523	0.807	0.843	0.863	0.559
	MF2	0.737	1.614				
	MF3	0.700	1.523				
	MF4	0.716	1.568				
	MF5	0.818	1.655				
SP	SP1	0.847	2.383	0.849	0.871	0.895	0.681
	SP2	0.839	3.325				
	SP3	0.856	3.508				
	SP4	0.757	1.292				

ANX, anxiety; FOC, fear of COVID-19; SP, student performance; MF, mindfulness.

**Figure 3:**
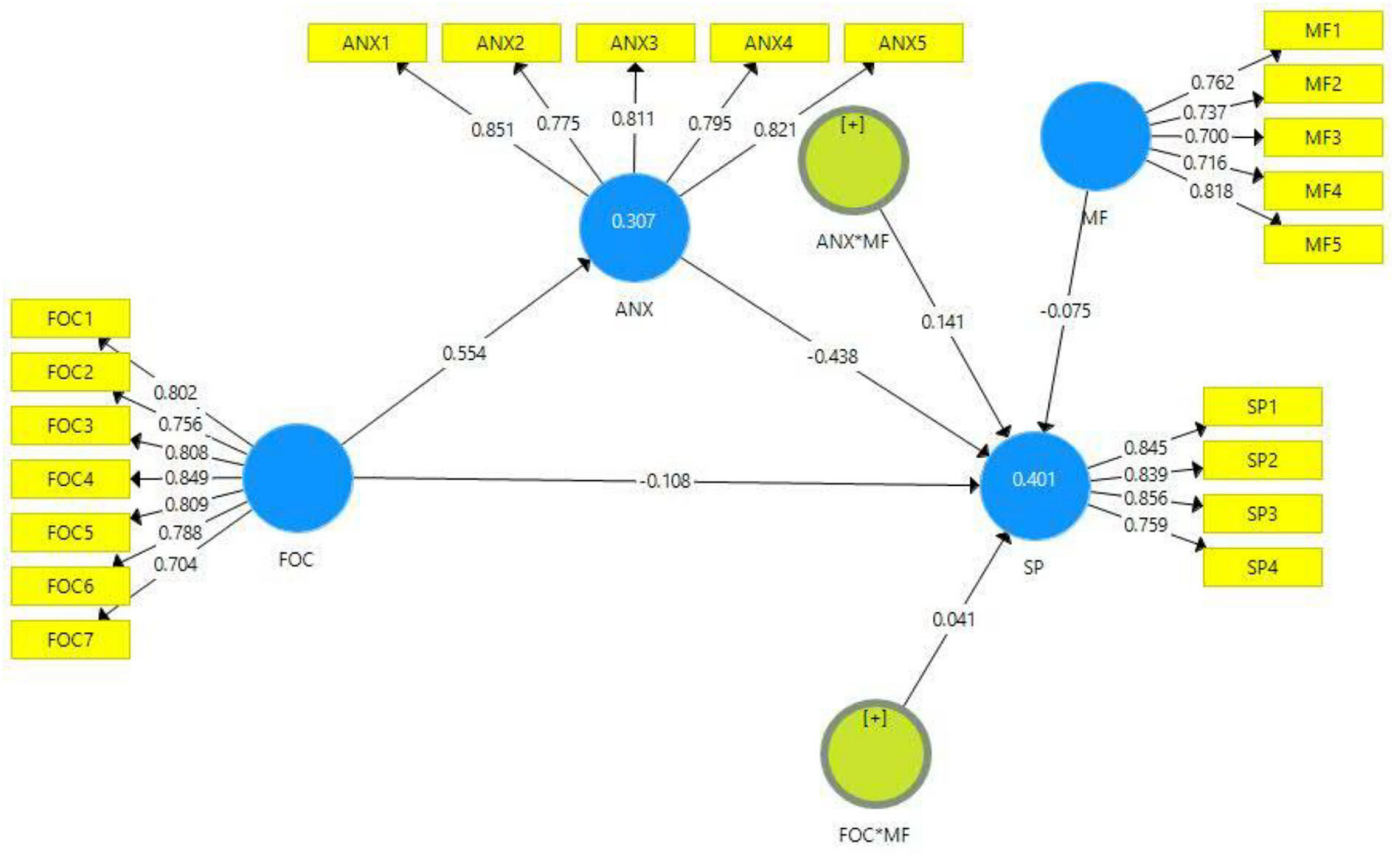
Path estimates and outer loadings (moderation).

The results of the moderation analysis confirmed the discriminant validity of the moderation effect (MF) through two approaches using Fornell–Larcker criterion and HTMT ratios. Tables 7, 8 describe the Fornell–Larcker criterion and HTMT ratios. The results show that the inner VIF values of all variables are significantly lower than 5 (Table 6), which demonstrates that there is no collinearity issue in the present study data. The *R*^2^ values of the endogenous variables of the current study's model (ANX and SP) re 0.307 and 0.401, respectively, which shows moderate model strength (79) (Figure 3).

**Table 7:** Discriminant validity (Fornell-Larker-1981 criteria) (moderation).

**Construct**	**ANX**	ANX^*^MF	**FOC**	FOC^*^MF	**MF**	**SP**
**ANX**	**0.811**					
ANX^*^MF	−0.513	**1.000**				
**FOC**	0.544	−0.395	**0.789**			
FOC^*^MF	−0.399	0.516	−0.372	**1.000**		
**MF**	0.231	−0.085	0.143	0.011	**0.748**	
**SP**	−0.604	0.435	−0.432	0.328	−0.202	**0.826**

ANX, anxiety; FOC, fear of COVID-19; SP, student performance; MF, mindfulness. Bold values indicate the relationship and significance between the variables.

* Relationship between variables.

**Table 8:** Discriminant validity (HTMT) (moderation).

**Construct**	**ANX**	ANX^*^MF	**FOC**	FOC^*^MF	**MF**	**SP**
**ANX**	–	**–**	**–**	**–**	**–**	**–**
ANX^*^MF	0.551	**–**	**–**	**–**	**–**	**–**
**FOC**	0.526	0.416	**–**	**–**	**–**	**–**
FOC^*^MF	0.429	0.516	0.392	**–**	**–**	**–**
**MF**	0.273	0.099	0.168	0.032	**–**	**–**
**SP**	0.626	0.447	0.475	0.353	0.210	**–**

ANX, anxiety; FOC, fear of COVID-19; SP, student performance; MF, mindfulness.

* Relationship between variables.

### Model estimation, moderation (stage 2)

The present study checked the moderating role of mindfulness between fear of COVID-19 and students' performance and the relationship between anxiety and students' performance, respectively. For empirical investigation, the current study assumed hypothesis 5 which outlined that mindfulness positively moderates the relationship between fear of COVID-19 and students' performance. However, the results (*t* = 0.052, *p* = 0.803) revealed that mindfulness does not moderate the relationship between fear of COVID-19 and students' performance (Table 9). Hence hypothesis 5 of the present study is rejected. The outcomes of hypothesis 6 (*t* = 2.33, *p* = 0.019) confirmed that mindfulness positively moderates the relationship between anxiety and students' performance; therefore, hypothesis 6 of the present study is accepted (Table 9).

**Table 9:** Hypotheses testing (moderation).

	**Moderation hypotheses**	**Coefficient (beta)**	**SD**	***t***	***p*-Value**	**Status**
H5	FOC*MF **→** SP	0.041	0.034	0.052	0.803	Not supported
H6	ANX^*^MF **→** SP	0.141	0.060	2.338	0.019	Supported

ANX, anxiety; FOC, fear of COVID-19; SP, student performance; MF, mindfulness.

* Relationship between variables.

**Figure 4:**
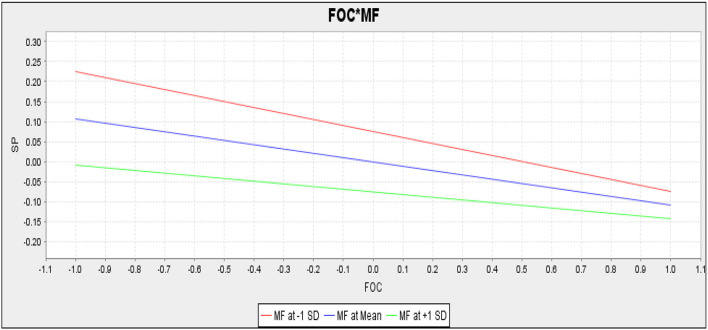
Slope for fear of COVID-19 (FOC) and mindfulness (MF) (moderation).

**Figure 5:**
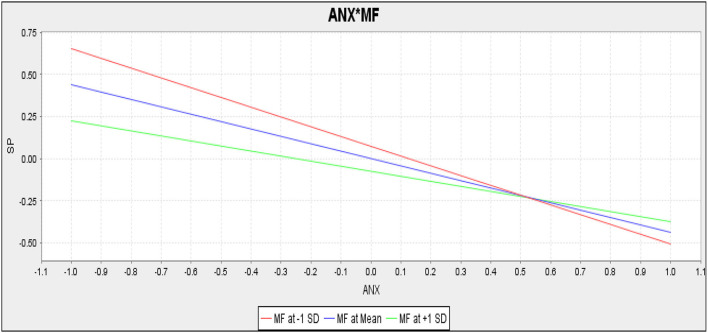
Slope for anxiety (ANX) and mindfulness (MF) (EHL) (moderation).

Mindfulness did not moderate the slope (Figure 4) reflecting the relationship between fear of COVID-19 and students' performance. Contrarily, mindfulness positively moderated the slope (Figure 5) reflecting the relationship between anxiety and students' performance.

## Discussion

The COVID-19 pandemic and its adverse consequences paved the way for fears, worries, anxiety, and uncertainties among individuals worldwide. It created difficulties and problems in almost everyone's daily life routine (84). People had to adjust their working patterns according to the turbulent pandemic situation. People felt uncertain about their future due to unpredictable deviations experienced during the epidemic. Organizations had to plan alternative schedules and strategies to execute their day-to-day work routine (85). Likewise, educational institutions were also forced to shift their academic activities from face-to-face to online platforms (86). These shifting study patterns required proper arrangements, tools, and tactics to deal with an evolving and dynamic situation. Online academic activities may have caused attitudinal and behavioral changes in students' learning patterns (87). With the support of stress theory, this study tried to determine how fear of COVID-19 impacted students' academic performance. For empirical investigation, this study assumed that fear of COVID-19 negatively affected students' performance. The study also assumed that fear of COVID-19 had a positive association with anxiety, and anxiety negatively affected students' performance. Moreover, this study also attempted to determine the mediating role of anxiety in the relationship between fear of COVID-19 and students' performance. Similarly, the study also attempted to check the moderating role of mindfulness between fear of COVID-19 and student performance and the relationship between anxiety and student performance.

The results of the present study show that hypothesis 1 was accepted, which proved that fear of COVID-19 had a negative association with students' performance. The findings are consistent with prior studies (88, 89), and according to these authors, the fear of being a victim of COVID-19 adversely impacted students' wellbeing and affected their academic performance. Moreover, the findings of this study confirmed that the fear of COVID-19 positively correlated with anxiety, which meant that hypothesis 2 was also accepted. Prior studies have demonstrated that fear of COVID-19 had adverse consequences on students' mental and psychological health (30, 49). Additionally, a study specifically acknowledged that fear of COVID-19 was a huge stumbling block in the students' learning process and led to anxieties and stresses of multiple kinds (90). Moreover, these adverse health conditions affect students' critical thinking abilities and academic performance. The findings proved that anxiety negatively affected students' performance which meant that hypothesis 3 of this study was accepted.

The current study revealed that anxiety negatively mediated the relationship between fear of COVID-19 and students' performance, confirming that hypothesis 4 was also accepted. These findings are consistent with similar other studies (2, 5). According to these studies, the fear of being a victim of COVID-19 adversely impacted students' wellbeing and critical thinking abilities. In addition, the fear of COVID-19 increased students' anxiety levels which in turn decreased their performance (91).

The present study also assumed the moderating role of mindfulness in the relationship between fear of COVID-19 and student performance. Similarly, it also sought to check the moderating role of mindfulness in the relationship between anxiety and student performance. The present study's findings reveal that hypothesis 5 was not accepted, which meant that mindfulness does not moderate the relationship between fear of COVID-19 and students' performance. Also, mindfulness may not positively moderate the relationship between fear of COVID-19 and student performance due to the disengagement and lack of self-efficacy of students (92). This study also found that mindfulness positively moderated the relationship between anxiety and student performance, which meant that hypothesis 6 was accepted. Previous research has also flagged that mindfulness could help individuals cope with anxiety and stress in their daily lives (93, 94). Moreover, it is understood that mindfulness can assist individuals in recovering from anxiety disorders. Mindfulness, therefore, can alleviate students' anxiety and positively impact their academic performance.

## Theoretical and practical implications

This research study has multiple theoretical and practical implications. Theoretically, this study adds valuable literature on the impact of fear of COVID-19 on students' academic performance and the mediating role of mindfulness in students with fear of COVID-19 and anxiety. The study fills the theoretical study gap on this particular research subject *via* literature review and novel data analysis. It is important to study these factors to improve the mental health status of HSK students and to enhance their academic performance by using mindfulness as a skill to overcome fear, stress, and anxiety. This study found that mindfulness positively mediated the relationship between fear of COVID-19 and HSK students' performance. It also positively moderated the relationship between anxiety and students' performance. Mindfulness reduces the fear of COVID-19 and anxiety and thus enhances students' academic performance. The literature provides extensive knowledge on mindfulness and mindfulness skills which can be used by students, teachers, and family members of the students and educationists to understand the use of mindfulness to boost the mental health of students in present times.

Furthermore, this study explains the positive association between fear of COVID-19 and anxiety. It also presents enough data and literature on the negative association of both of these parameters with the academic performance of students. The fear of COVID-19 negatively affects the academic performance of the students. It increases anxiety in students, which ultimately leads to stress and low academic performance. This study has explained the variables through the lens of stress theory; it adds to the literature on stress theory and how stress affects mental health by increasing fear and anxiety, which ultimately affects the students' academic performance. The study can be used practically to study variables like fear of COVID-19, anxiety, mindfulness, and student performance.

Along with theoretical implications the current study has many relevant and realistic practical implications as well. The knowledge and understanding obtained by this literature can be practically used by experts and other scientists to bring a practical change in the mental health of students by incorporating mindfulness skills into students' life and routines, which can promote their academic performance. This study has practical implications by offering an example for relevant research studies on other research subjects. It can be used to make desired changes in the mode of teaching and curriculum keeping in view the status of fear, anxiety, and stress to enhance the mental health of students. One of the most important practical implications of this study is to help design “academic support” using direct or indirect socializing sources for such students.

## Limitations

Like other studies of social sciences, this study also has some limitations, which could be opportunities for scholars to research in the future. First, this study was conducted with small sample size; future studies may increase the sample size to verify the present study's model. Second, this study used the structured questionnaire method for data collection; in the future, researchers may consider other methods of data collection for better response, such as semi-structured questionnaires, open-ended questionnaires, and interview methods. Third, this study was conducted in China, and the results cannot be generalized; scholars in the future may conduct the same study by considering other developing or developed countries to understand the study model in a better way. Fourth, this study examined the mediating role of anxiety; in the future, the researchers may consider other mental disorders like depression. Finally, this study took mindfulness as a moderator; however, future research may assess the moderating role of other factors such as self-efficacy and emotional intelligence in validating this study's model.

## Conclusion

The outbreak of COVID-19 and its adverse consequences have led to fears, anxiety, and uncertainties among individuals worldwide. Almost everyone has had to face some difficulties and challenges in daily life due to the pandemic. Educational institutions also had to shift their academic activities to online platforms, which in turn caused attitudinal and behavioral changes in students' learning patterns. Using stress theory, the present study attempted to determine the association of fear of COVID-19 with students' performance. In addition, the present study also tried to determine the impact of fear of Covid-19 on anxiety. The study assumed that anxiety negatively correlated with students' performance, and also assumed the mediating role of anxiety in the relationship between fear of COVID-19 and students' performance. Similarly, the study aimed to check the moderating role of mindfulness between fear of COVID-19 and student performance and assumed the moderating role of mindfulness in the relationship between anxiety and student performance. This study's findings confirmed that fear of COVID-19 negatively affected students' performance and positively correlated with anxiety. The study's findings also revealed that anxiety negatively affected students' performance and confirmed that anxiety negatively mediated the relationship between fear of COVID-19 and students' performance. However, the study's findings revealed that mindfulness did not moderate the relationship between fear of COVID-19 and student performance. On the other hand, the findings confirmed that mindfulness positively moderated the relationship between anxiety and student performance.

## Data availability statement

The original contributions presented in the study are included in the article/Supplementary material, further inquiries can be directed to the corresponding author.

## Ethics statement

The studies involving human participants were reviewed and approved by Shandong University of Finance and Economics, China. The patients/participants provided their written informed consent to participate in this study. The study was conducted in accordance with the Declaration of Helsinki.

## Author contributions

YL: conceptualization and data collection. ZM: writing the draft. Both authors agreed to the submitted version of the manuscript. Both authors contributed to the article and approved the submitted version.

## Funding

This work was funded by the Major Project of 2020 National Social Science Foundation: “Research on The Language of Social Governance in Cyberspace”.

## Conflict of interest

The authors declare that the research was conducted in the absence of any commercial or financial relationships that could be construed as a potential conflict of interest.

## Publisher's note

All claims expressed in this article are solely those of the authors and do not necessarily represent those of their affiliated organizations, or those of the publisher, the editors and the reviewers. Any product that may be evaluated in this article, or claim that may be made by its manufacturer, is not guaranteed or endorsed by the publisher.
